# Yezo Virus Diversity in Tick Bite Patients and Ticks, Russia

**DOI:** 10.3201/eid3205.251620

**Published:** 2026-05

**Authors:** Yulia O. Epik, Kseniia A. Sycheva, Lyudmila S. Karan, Evgenia V. Mokretsova, Anna G. Dragomeretskaya, Olga E. Trotsenko, Anna R. Efimova, Yulia M. Spirina, Olga M. Drozdova, Tatyana E. Bondarenko, Ekaterina A. Blinova, Vasiliy G. Akimkin, Evgeny S. Morozkin

**Affiliations:** Central Research Institute of Epidemiology, Moscow, Russia (Y.O. Epik, K.A Sycheva, E.A. Blinova, V.G. Akimkin, E.S. Morozkin); Russian Сenter of Neurology and Neurosciences, Moscow (L.S. Karan); Far Eastern State Medical University, Khabarovsk, Russia (E.V. Mokretsova); Khabarovsk Research Institute of Epidemiology and Microbiology, Khabarovsk (E.V. Mokretsova, A.G. Dragomeretskaya, O.E. Trotsenko), Centre of Hygiene and Epidemiology in the Kemerovo region–Kuzbass, Kemerovo, Russia (A.R. Efimova, Y.M. Spirina); Kemerovo State Medical University, Kemerovo (O.M. Drozdova); Kuzbass Clinical Infectious Diseases Hospital, Kemerovo (T.E. Bondarenko).

**Keywords:** Yezo virus, *Orthonairovirus*, *Orthonairovirus yezoense*, tick-borne, tick-borne infections, vector-borne infections, viruses, *Ixodes persulcatus*, molecular diagnosis, Russia

## Abstract

Yezo virus is an emerging tickborne orthonairovirus. We detected Yezo virus RNA in tick bite patients and in *Ixodes persulcatus* ticks in west Siberia and Far East, Russia. Clinicians should consider the expanded area of Yezo virus identification and the *I. persulcatus* tick habitat when evaluating patients after tick bites in Russia.

Tickborne orthonairoviruses (Nairoviridae, *Orthonairovirus*) are a recognized global health threat to humans. In Russia, the most common pathogenic agent to humans within the genus *Orthonairovirus* is Crimean-Congo hemorrhagic fever virus, transmitted by ticks belonging to the family Ixodidae. Crimean-Congo hemorrhagic fever virus is endemic to southern regions in Russia and causes >100 annual severe fever cases in humans (*1*).

In recent years several novel orthonairoviruses have been discovered in China and Japan. Songling virus (*2*), Tacheng tick virus 1 (*3*), and Beiji nairovirus (*4*) are associated with human febrile illness in northeastern and northwestern China. Yezo virus (YEZV) (*Orthonairovirus yezoense*), a newly described tickborne orthonairovirus, was identified in Japan in 2021 and causes acute febrile illness accompanied by thrombocytopenia and leukopenia. YEZV was detected in blood samples from 7 patients in Hokkaido, Japan (*5*), and 1 patient in northeastern China (*6*) after tick bites. Afterward, 18 tick bite patients with YEZV infection were identified in northeastern China (*7*). Furthermore, YEZV was identified in ixodid ticks in both countries. We report detection of YEZV in *Ixodes persulcatus* ticks and the serum of tick bite patients in Russia.

## The Study

We retrospectively investigated the presence of YEZV RNA in 310 blood samples from tick bite patients. The samples included 144 samples from patients hospitalized in the Kemerovo region of Russia during 2024 and 2 sets of samples from the Khabarovsk region, 60 samples from patients hospitalized in 2015 and 106 samples from patients hospitalized in 2023–2024. We used an in-house real-time reverse transcription PCR (qRT-PCR) for YEZV RNA detection (Appendix Table 1). We identified 2 positive samples: 1 from the Kemerovo region of Russia and 1 from the Khabarovsk region of Russia.

The patient from the Khabarovsk region, Far East, Russia, was a 68-year-old woman who was bitten by a tick in 2015; the species of tick was not determined. Five days after the bite, tick bite fever developed, accompanied by headache, malaise, myalgia, lumbago, and nausea. Two days after fever onset, the patient was hospitalized. A physical examination at admission revealed no meningeal symptoms or rash (Table 1). An eschar, ≈4 mm in diameter and consistent with a tick bite site, was observed. Laboratory testing did not detect thrombocytopenia or leukopenia. Serum levels of liver aminotransferases were unremarkable. While hospitalized, the patient received unidox solutab (100 mg 2×/d for 8 d) (Appendix Table 2). The patient fully recovered on day 9 of hospitalization and was discharged. Molecular investigation of the patient’s blood samples revealed negative results for common tickborne pathogens. However, positive results were obtained for *Rickettsia heilongjiangensis* and YEZV (Table 1). qRT-PCR analysis detected YEZV RNA at a concentration of 10^6^ copies/mL in the patient’s serum collected on day 1 after fever onset.

**Table 1:** Characteristics of 2 Yezo virus–infected patients with history of tick bites from Khabarovsk and Kemerovo regions, Russia

Epidemiologic characteristics	Patient 1	Patient 2
Age, y	68	69
Sex	F	M
Geographic region	Khabarovsk	Kemerovo
Days from tick bite to symptom onset	5	3
Days from symptom onset to hospital admission	2	0
Tick bite identification method	Self-reported	Self-reported
Clinical manifestations		
Fever	Yes	Yes
Headache	Yes	No
Malaise	Yes	Yes
Nausea	Yes	No
Myalgia	Yes	Yes
Lumbago	Yes	No
Rash	No	No
Meningeal symptoms	No	No
Bacterial co-infection	Yes	No
Tickborne pathogen detection		
Tickborne encephalitis virus	No	No
*Borrelia* spp.	No	No
*Anaplasma* spp.	No	No
* Ehrlichia chaffeensis*	No	No
* Ehrlichia muris*	No	No
* Rickettsia heilongjiangensis*	Yes	No
Yezo virus	Yes	Yes

The second clinical case occurred in the Kemerovo region, West Siberia, Russia, in 2024. The patient, a 69-year-old man, was admitted to the hospital with a diagnosis of tickborne fever of moderate severity. His clinical manifestation included fever, malaise, and myalgia (Table 1). The patient reported a tick bite 3 days earlier; however, the tick was not available for examination. Platelet and leukocyte counts and serum levels of liver aminotransferases were unremarkable. While hospitalized, the patient received prophylactic intramuscular ceftriaxone (1 g 2×/d for 9 d) (Appendix Table 2). He was discharged on day 9 after a full recovery. Molecular analysis of his blood sample for tickborne pathogens yielded negative results (Table 1). qRT-PCR analysis detected YEZV RNA at a concentration of 5 × 10^5^ copies/mL in serum collected on the first day after fever onset.

To assess the epidemiologic situation in Russia, we tested a total of 2,497 ticks, consisting of 3 species (*I. persulcatus*, *Haemaphysalis concinna*, and *H*. *japonica*), collected during 2006–2023 in the Kemerovo region and during 2023 from the Khabarovsk region of Russia (Table 2). We tested for YEZV RNA testing by using qRT-PCR. We amplified positive samples by using in-house primer pairs (Appendix Table 3) and then performed sequencing.

**Table 2:** Detection of Yezo virus RNA in ixodid ticks from Khabarovsk and Kemerovo regions, Russia*

Region	Sampling year	Tick species	No. ticks	No. pools	No. positive pools	Prevalence	SE
Kemerovo	2006	*Ixodes persulcatus*	700	70	6	0.9%	0.0036
	2023	*I. persulcatus*	648	216	2	0.3%	0.0022
Khabarovsk	2023	*I. persulcatus*	816	272	4	0.5%	0.0025
	2023	*Haemaphysalis concinna*	159	53	0	NA	NA
	2023	*H. japonica*	168	56	0	NA	NA
Total			2,491	667	12		

*We calculated the prevalence by using Epitools (https://epitools.ausvet.com.au). NA, not available.

We detected YEZV RNA exclusively in *I. persulcatus* ticks from both the Kemerovo and Khabarovsk regions of Russia (Figure). The prevalence of YEZV infection in ticks was determined to be 0.5% in the Khabarovsk region and ranged from 0.3% to 0.9% in the Kemerovo region in different sampling years (Table 2).

**Figure:**
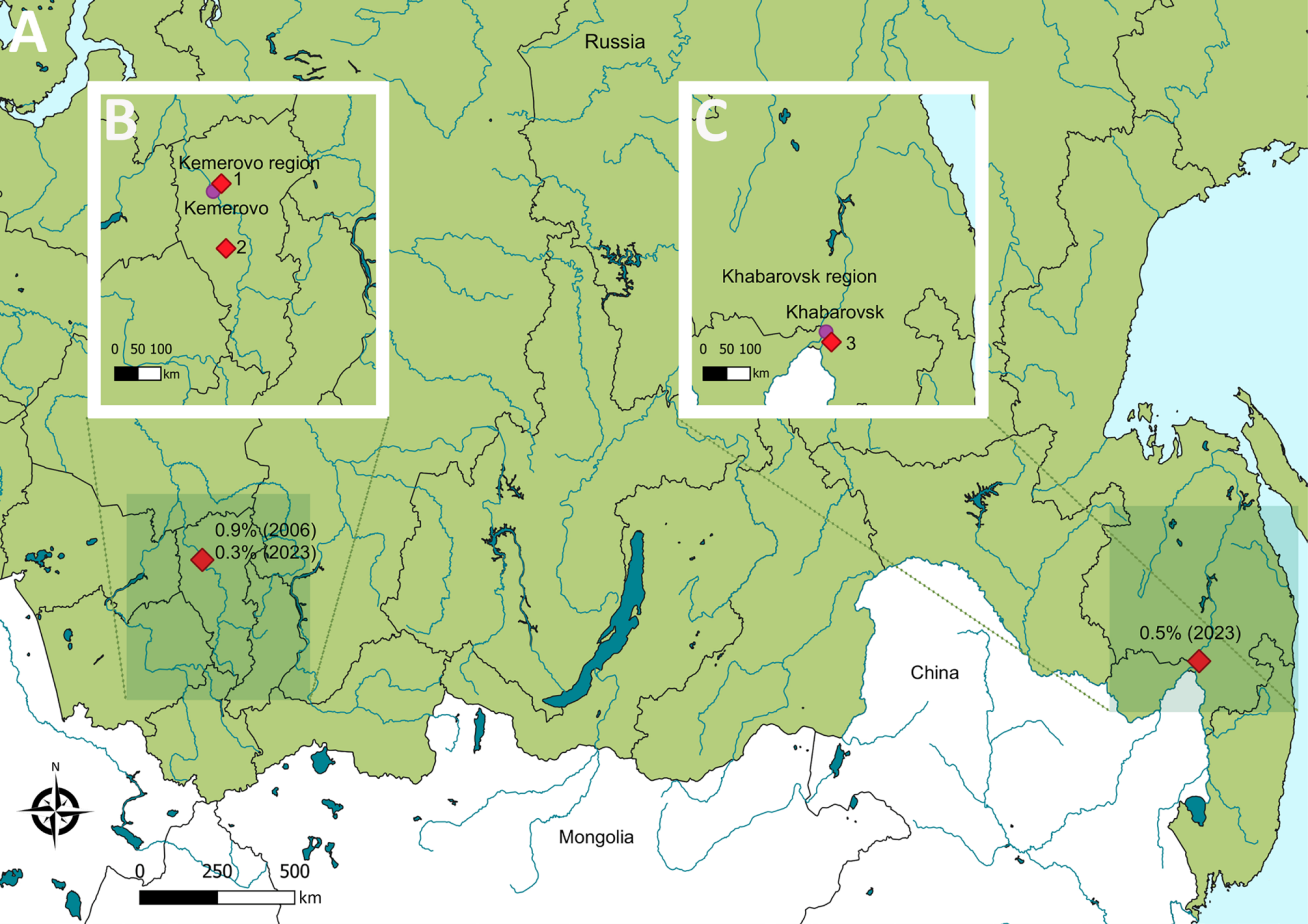
Geographic distribution of Yezo virus (YEZV) detection in ticks from Russia. Red diamonds indicate sampling locations of YEZV-positive *Ixodes persulcatus* ticks. A) Distribution across Russia. YEZV prevalence (%) and sampling year are indicated adjacent to symbols. B) Distribution within the Kemerovo region. Samples of isolates 143 (GenBank accession nos. PV770292, PX898221, and PX904396), 24-4 (accession nos. PV770294, PX898220, and PX904395), 6-1 (accession nos. PV770295 and PX898217), 6-4 (accession nos. PV770296 and PX898218), and 6-5 (accession nos. PV770297, PX898219, and PX904394) were derived from ticks collected in location 1. The sample of isolate 165 (accession nos. PV770293, PX898222, and PX904397) was derived from a tick collected in location 2. C) Distribution within the Khabarovsk region. Samples of isolates 560 (accession nos. PV770287, PX898225, and PX904391), 576 (accession nos. PV770288, PX898226, and PX904392), 649 (accession nos. PV770289, PX898227, and PX904390), and 662 (accession nos. PV770290, PX898228, and PX904393) were derived from ticks collected in location 3.

We obtained 12 nucleoprotein, 12 glycoprotein precursor gene segment, and 10 RNA-dependent RNA polymerase sequences of YEZV. In addition to the obtained sequences, we included sequences from China, Japan, and Russia, accessed from GenBank, in the analysis. We selected Sulina virus, the closest known relative of YEZV, as the outgroup. Our analysis revealed the obtained sequences formed 3 distinct clades across all 3 genome segments: 1 from the Khabarovsk region (clade A) and 2 from the Kemerovo region (clades B and C) (Appendix Figure 1). Khabarovsk variants exhibited close phylogenetic proximity to sequences from China and Japan. Nucleotide differences between sequences from clades B and C (Kemerovo region) were 7.8% for the nucleoprotein gene segments, 9.0% for the glycoprotein precursor gene segments, and 9.7% for the RNA-dependent RNA polymerase gene segments (Appendix Figure 2). Of note, the division of viruses from Kemerovo sequence into 2 distinct clades correlated with their specific tick sampling locations. All viral genome sequences from this study have been deposited into GenBank (accession nos. PV770287–98, PX898217–28, PX904390–9).

## Conclusions

We identified 3 genomic variants of YEZV within Russia. The variant from Khabarovsk is closely related to viruses previously reported in China and Japan. We detected 2 genomic variants in the Kemerovo region. This genetic diversity, which correlates with sampling location, might indicate an association between different YEZV variants and distinct *I. persulcatus* tick populations (*8*). Reassortment events in the YEZV genome have previously been reported (strains BT-2135 and T-HLJ02) (Appendix Figure 1 (*9*); however, we did not detect reassortant strains among the obtained sequences. Further investigation is needed to determine the causes of high diversity of YEZV in West Siberia, Russia, compared with Japan and China, and to identify possible novel genetic variants of the YEZV by studying more western regions of the *I. persulcatus* tick habitat.

We identified 2 patients with YEZV infection after tick bites: 1 from the Kemerovo region with monoinfection and the other from the Khabarovsk region with co-infection with *R. heilongjiangensis*. Both patients’ clinical manifestations included nonspecific symptoms common to many infections. Because neither patient had traveled abroad, the cases represent autochthonous YEZV transmission within Russia. All blood samples were tested for the presence of YEZV RNA retrospectively, preventing the investigation of blood parameter dynamics. Previous studies have reported that, in addition to general clinical manifestations such as fever and malaise, YEZV infection might be accompanied by elevated serum levels of liver aminotransferases (alanine aminotransferase and aspartate aminotransferase), thrombocytopenia, and leukopenia (*5*–*7*,*10*). In both cases we identified, platelet and leukocyte counts and liver aminotransferase levels were unremarkable (Appendix Table 2).

Specific clinical manifestations of YEZV infection that would enable reliable clinical diagnosis are difficult to define. Other common tickborne diseases, such as tick-borne encephalitis, borreliosis, and rickettsiosis, do not always occur with specific symptoms, which can lead to misdiagnosis. In those cases, qPCR or qRT-PCR is used to confirm the etiologic agent. The emergence of YEZV as a novel tickborne pathogen highlights the need to update diagnostic panels for routine testing of patients with unclear clinical manifestations after tick bites.

Appendix Additional information on Yezo virus diversity in tick bite patients and ticks, Russia.
