# Supplementary material for: Yezo Virus Diversity in Tick Bite Patients and Ticks, Russia
Source: Emerg Infect Dis. 2026 May;32(5):759–62. doi: 10.3201/eid3205.251620 (PMC13175110; doi:10.3201/eid3205.251620)
Supplement: Appendix — Additional information on Yezo virus diversity in tick bite patients and ticks, Russia. [file 25-1620-Techapp-s1.pdf]

Appendix DOI: https://doi.org/10.3201/eid3205.251620

EID cannot ensure accessibility for supplementary materials supplied by authors. Readers who have difficulty accessing supplementary content should contact the authors for assistance.

Yezo Virus Diversity in Tick Bite Patients and Ticks, Russia

Appendix.

Experimental workflow used in this study.

Ticks were collected using the flagging method. Species identification was conducted under a stereomicroscope based on morphological features. For additional verification an in-house qPCR assay was used to distinguish between two morphologically similar species: *Ixodes persulcatus* and *Ixodes pavlovskyi*, and their hybrids (Appendix Table 4). The collected ticks were washed once in 70% ethanol and twice in 0.15 M NaCl solution. Individual ticks were homogenized in 300 µl of 0.15 M NaCl solution and pooled into groups of 3–10 based on species, sex, and sampling location. Total RNA was extracted from 100 µl of the pooled tick suspension using the RIBO-Prep kit (AmpliSens, Moscow, Russia) and from 1 ml of patient's serum using the MAGNO-Sorb kit (AmpliSens, Moscow, Russia). The extracted RNA was screened for Yezo virus RNA using the in-house two-step RT-qPCR assay (Appendix Table 1). Primers and a probe for the RT-qPCR assay were designed based on an alignment of all Yezo virus sequences available in GenBank. The sensitivity of the assay was determined using a recombinant control and was estimated to be 10 copies per reaction. The specificity of the assay was evaluated using RNA of other orthonairoviruses, including Sulina virus, Tacheng tick virus 1, Crimean–Congo hemorrhagic fever virus, and Beiji nairovirus. In the first step, RNA was reverse transcribed into cDNA using the Reverta-L kit (AmpliSens, Moscow, Russia). In the second step, qPCR was performed under the following thermal cycling conditions: 95°C for 15 min; 45 cycles of 95°C for 10 s, 58°C for 30 s, 72°C for 10 s. The fluorescence signal was detected by a Rotor-Gene amplifier (Qiagen, Germany).

The concentration of viral RNA copies in the blood of Yezo-infected patients was estimated using a dilution series of the recombinant plasmid. The prevalence of Yezo virus in

ticks was calculated using the Epitools online calculator (https://epitools.ausvet.com.au/), specifically the “Pooled prevalence for fixed pool size and perfect tests, Method 2” tool. The map illustrating YEZV prevalence in ticks across Russian regions was created using QGIS software (QGIS Development Team, http://qgis.osgeo.org).

The genome of YEZV-positive samples was amplified using in-house primers (Appendix Table 3). cDNA libraries for high-throughput sequencing were constructed from S segment of four samples from the Khabarovsk region, from M segment of twelve samples and from L segment of ten samples using the Nextera XT DNA Library Preparation Kit (Illumina, USA). Paired-end sequencing was performed on an Illumina MiSeq sequencer (Illumina, USA). Raw reads were subjected to quality control, including adaptor trimming and removal of low-quality sequences. This was performed using Trimmomatic (v0.39) with the parameters LEADING:7, TRAILING:7, SLIDINGWINDOW:4:15, MINLEN:40, and the ILLUMINACLIP option specifying the adaptor sequence file. Consensus genome assembly was carried out de novo using bowtie2 (v2.4.4). The S segments of the remaining eight samples were Sanger sequenced. Primary sequencing data were analyzed using Vector NTI software version 10.3.0. Sequences identified in this study were deposited in the GenBank database (Appendix Table 5). Obtained sequences and sequences of published Yezo viruses were downloaded from the National Center for Biotechnology Information (NCBI) public databases (https://www.ncbi.nlm.nih.gov) and aligned together using MEGA version 12 (https://www.megasoftware.net). The maximum-likelihood tree of S segment was inferred with MEGA under Tamura and Nei 1993 model with gamma rate categories and invariable sites (TN93+G+I) based on the minimum Bayesian Information Criterion (BIC) score with 1,000 bootstrap replicates. The maximum-likelihood tree of M segment was inferred with MEGA under General Time Reversible model with gamma rate categories and invariable sites (GTR+G+I) based on the minimum Bayesian Information Criterion (BIC) score with 1,000 bootstrap replicates. The maximum-likelihood tree of L segment was inferred with MEGA under General Time Reversible model with invariable sites (GTR+I) based on the minimum Bayesian Information Criterion (BIC) score with 1,000 bootstrap replicates. The phylogenetic tree was visualized in the Interactive Tree Of Life (iTOL) version 7 (https://itol.embl.de/).

Appendix Table 1. Primer sequences used in this study for Yezo virus RT-qPCR detection targeting the fragment of S segment.

Primer name	Sequence (5′→3′)
Yezo-For	GCCTACAAGTGGGGAAGCAC
Yezo-Rev	CTTAACAGGGTTTGACAGAGGGA
Yezo-Probe	ROX-CACTCCCCACAGAATGTCTGAGATG-BHQ2

Appendix Table 2. Laboratory findings of two tick-bitten Yezo virus infected patients from Khabarovsk and Kemerovo regions, Russia

All characteristics	Patient 1 (reference range)	Patient 2 (reference range)
Epidemiologic characteristics		
Age (years)	68	69
Sex	Female	Male
Geographic region	Khabarovsk	Kemerovo
Days after symptoms onset	3	3
Laboratory results		
Platelets, 10^9/L	256 (179–403)	189 (187–381)
Leukocytes, 10^9/L	4.7 (4.5–11)	8 (4.5–11)
Lymphocytes, %	30 (19–37)	19.8 (19–37)
Red blood cells, 10^{12}/L	3.8 (3.8–5.2)	4.4 (3.8–5.8)
Hemoglobin, g/L	117 (117–161)	136 (126–174)
Urea nitrogen, mmol/L	5.1 (2.6–6.8)	7.9 (2.9–8.2)
Aspartate aminotransferase, U/L	30 (<35)	36.2 (<50)
Alanine aminotransferase, U/L	34 (<35)	17.4 (<50)
Creatinine, µmol/L	61 (49–90)	84.9 (64–104)
Total bilirubin, µmol/L	5.4 (5–21)	5.9 (5–21)
Treatment during hospitalization*	Unidox-SoluTab (100 mg, orally, 2×/d), Ketorolac (1 ml, IM, 2×/d)	Ceftriaxone (1 g, IM, 2×/d); Ibuprofen (400 mg, orally, 1×/d)

*IM, intramuscular

Appendix Table 3. Primer sequences used in this study for Yezo virus genome amplification.

Primer	Nucleotide Sequence (5′–3′)	Amlicon length, bp	Target
YezoL-F-25	CCCCCACTAAGGMTAACCTC	1319	L segment
YezoL-R-1320	TCTCTTCTCAACCATTCTACTCT		
YezoL-F-1230	TCATCAGCCGCCATGCTGGCT	1219	
YezoL-R-2430	GACTCTTCCAAGTCATGTGCT		
YezoL-F-2250	ARCCGACACTGAGCATGATAGT	1231	
YezoL-R-3460	GTTAGTTCAGAGTCATCTGTGCT		
YezoL-F-3340	TGTCTGAACACCTGCCATGTTG	1311	
YezoL-R-4630	AGGTGTTGACTATCATGTCGTAACT		
YezoL-F-4450	AGAGGTGCTTAGATGAACTGACT	1310	
YezoL-R-5730	TATCTTGTCTTTGCCCTGCTCCA		
YezoL-F-5610	GACATCCATTGTGTCTGAGCCA	1114	
YezoL-R-6700	AGTATTYACAGCCTCATCAGGCT		
YezoL-F-6580	AATGGACTTTCACCGCCAGATG	1169	
YezoL-R-7730	TCATCAGAGCTGCCTGCATGT		
YezoL-F-7590	ACATGTTACAACCACATGGGCCA	1213	
YezoL-R-8780	TCATTGGGTCCTCTATGACTCT		
YezoL-F-8640	GAAACTGCTGGAAGARAAGCCCA	1196	
YezoL-R-9810	TGGGTTCTGTGTCAGTTGCCA		
YezoL-F-9650	ACAGTRTGCAACAGCATCACAGA	1179	
YezoL-R-10810	TCTRATCTTTCCTATCACGACCT		
YezoL-F-10620	AAGAGCAGCAAGACCTGTGACT	1512	
YezoL-R-12100	CTGCATACCCCCCTATTATAACCT		
YezoM-F-38	ACCTCACTGGTTGYTAGGACC	1218	M segment
YezoM-R-1230	CTTAGAGTGTGCAAATGATGACTCT		
YezoM-F-1150	CACAACCATGCCCACCAGAC	1071	
YezoM-R-2200	ACAGTACTCTTCCTCATACACACA		
YezoM-F-2060	ACCTTGAGCAGAATTCAGTGGGT	1181	
YezoM-R-3210	CAATCACCAGGGTTGCAGTAGT		
YezoM-F-3050	TGCAAGCTTCAGGCATGCACA	1061	
YezoM-R-4100	CTCAGCAGTTCCTCTTCCTCT		
YezoS-F-45	ACCGGAGATGGCACGTCTGA	828	S segment
YezoS-R-850	TCATCCATCTTCTGCTGGTTGT		
YezoS-F-675	GTGAAAGGAGGTGAAGATAGGA	1014	
YezoS-R-1670	TGTTGTTGCTGCATACCCCCT		

Appendix Table 4. Primer sequences used in this study for differentiation between *Ixodes persulcatus* and *Ixodes pavlovskyi* via qPCR targeting the fragment of cytochrome c oxidase I (CO1) gene.

Primer name	Sequence (5′→3′)
I.pers-F	AAAGAGGAGCAGGGACAGGA
I.pers-R	GCTATATCRACTGATGCACCT
I.pers-Pr	R6G-CTGTTTATCCTCCTCTATCATCTAACATC-BHQ1
I.pavl-F	AGAGAGGAGCAGGAACAGGA
I.pavl-R	GCTATATCAACAGAAGCACCT
I.pavl-Pr	ROX-CAGTCTATCCCCCRCTTTCATCTAATATC-BHQ2

Appendix Table 5. Information of YEZV partial S, M, and L segments sequences identified in this study.

Genbank accession number	Isolate	Segment	Sample collection region	Host	Collection date
PV770287	560	S	Khabarovsk	*Ixodes persulcatus*	2023 Jun
PV770288	576	S	Khabarovsk	*Ixodes persulcatus*	2023 Jun
PV770289	649	S	Khabarovsk	*Ixodes persulcatus*	2023 Jun
PV770290	662	S	Khabarovsk	*Ixodes persulcatus*	2023 Jun
PV770291	7	S	Kemerovo	*Homo sapiens*	2024 Jun
PV770292	143	S	Kemerovo	*Ixodes persulcatus*	2023 Apr
PV770293	165	S	Kemerovo	*Ixodes persulcatus*	2023 Apr
PV770294	24–4	S	Kemerovo	*Ixodes persulcatus*	2006 Apr
PV770295	6–1	S	Kemerovo	*Ixodes persulcatus*	2006 Apr
PV770296	6–4	S	Kemerovo	*Ixodes persulcatus*	2006 Apr
PV770297	6–5	S	Kemerovo	*Ixodes persulcatus*	2006 Apr
PV770298	180	S	Khabarovsk	*Homo sapiens*	2015 Jun
PX898217	6–1	M	Kemerovo	*Ixodes persulcatus*	2006 Apr
PX898218	6–4	M	Kemerovo	*Ixodes persulcatus*	2006 Apr
PX898219	6–5	M	Kemerovo	*Ixodes persulcatus*	2006 Apr
PX898220	24–4	M	Kemerovo	*Ixodes persulcatus*	2006 Apr
PX898221	143	M	Kemerovo	*Ixodes persulcatus*	2023 Apr
PX898222	165	M	Kemerovo	*Ixodes persulcatus*	2023 Apr
PX898223	7	M	Kemerovo	*Homo sapiens*	2024 Jun
PX898224	180	M	Khabarovsk	*Homo sapiens*	2015 Jun
PX898225	560	M	Khabarovsk	*Ixodes persulcatus*	2023 Jun
PX898226	576	M	Khabarovsk	*Ixodes persulcatus*	2023 Jun
PX898227	649	M	Khabarovsk	*Ixodes persulcatus*	2023 Jun
PX898228	662	M	Khabarovsk	*Ixodes persulcatus*	2023 Jun
PX904390	649	L	Khabarovsk	*Ixodes persulcatus*	2023 Jun
PX904391	560	L	Khabarovsk	*Ixodes persulcatus*	2023 Jun
PX904392	576	L	Khabarovsk	*Ixodes persulcatus*	2023 Jun
PX904393	662	L	Khabarovsk	*Ixodes persulcatus*	2023 Jun
PX904394	6–5	L	Kemerovo	*Ixodes persulcatus*	2006 Apr
PX904395	24–4	L	Kemerovo	*Ixodes persulcatus*	2006 Apr
PX904396	143	L	Kemerovo	*Ixodes persulcatus*	2023 Apr
PX904397	165	L	Kemerovo	*Ixodes persulcatus*	2023 Apr
PX904398	180	L	Khabarovsk	*Homo sapiens*	2015 Jun
PX904399	7	L	Kemerovo	*Homo sapiens*	2024 Jun

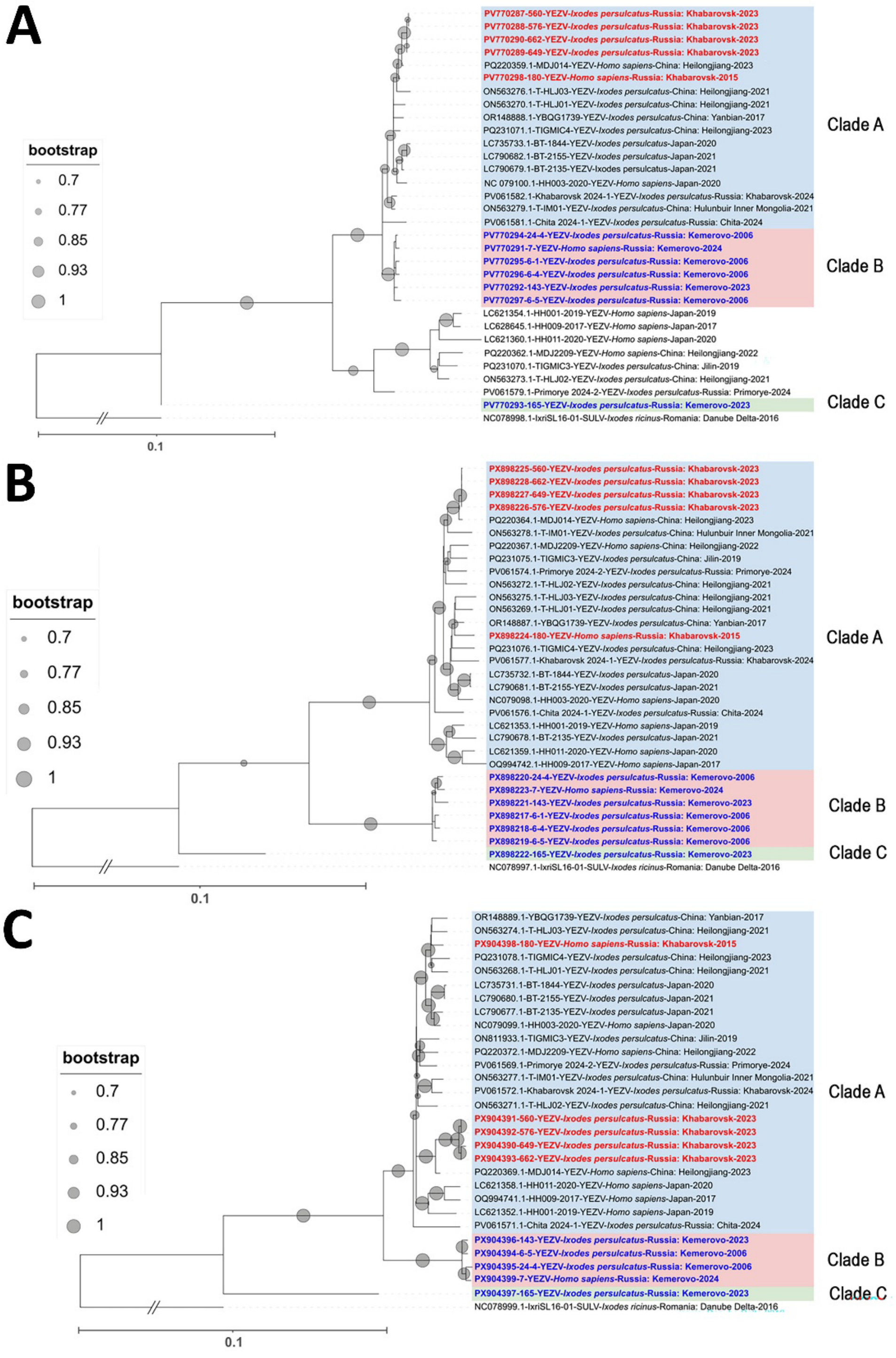

Appendix Figure 1. Phylogenetic analyses of Yezo virus S, M, and L segments from tick-bitten patients and *Ixodes persulcatus* ticks. A) S segment tree based on partial open reading frame (ORF) of 1,436 nt; B) M segment tree based on partial ORF of 3,895 nt; C) L segment tree based on partial ORF of 5,471 nt. Sequences identified in study of Yezo virus diversity in tick-bitten patients and ticks in Russia are indicated in color: sequences from the Khabarovsk region (red) and from the Kemerovo region (blue).

Sequences of previously published Yezo viruses were downloaded from National Center for Biotechnology Information public databases (https://www.ncbi.nlm.nih.gov) and aligned together using MEGA version 12 (https://www.megasoftware.net). The maximum-likelihood tree was inferred with MEGA v.12 under the TN93+G+I model for S segment, the GTR+G+I model for M segment, the GTR+I model for L segment with 1,000 bootstrap replicates. Nodes with bootstrap support >0.7 are denoted by gray circles. Three distinct clades containing sequences obtained in this study are highlighted in blue (Clade A), red (Clade B), and green (Clade C).

A

		1	2	3	4	5	6	7	8	9	10	11	12	13	14	15	16	17	18	19	20	21	22	23	24	25	26	27	28	29	30	31
1	PV770287-560-YEZV-Ixodes_persulcatus-Russia:_Khabarovsk-2023																															
2	PV770288-576-YEZV-Ixodes_persulcatus-Russia:_Khabarovsk-2023	0,00%																														
3	PV770290-662-YEZV-Ixodes_persulcatus-Russia:_Khabarovsk-2023	0,07%	0,07%																													
4	PV770289-649-YEZV-Ixodes_persulcatus-Russia:_Khabarovsk-2023	0,07%	0,07%	0,00%																												
5	PQ220359.1-MDJ014-YEZV-Homo_sapiens-China:_Heilongjiang-2023	0,35%	0,35%	0,28%	0,28%																											
6	PV770298-180-YEZV-Homo_sapiens-Russia:_Khabarovsk-2015	0,49%	0,49%	0,42%	0,42%	0,28%																										
7	ON563276.1-T-HLJ03-YEZV-Ixodes_persulcatus-China:_Heilongjiang-2021	1,04%	1,04%	0,97%	0,97%	0,84%	0,70%																									
8	ON563270.1-T-HLJ01-YEZV-Ixodes_persulcatus-China:_Heilongjiang-2021	1,11%	1,11%	1,04%	1,04%	0,91%	0,77%	1,11%																								
9	OR148888.1-YBQG1739-YEZV-Ixodes_persulcatus-China:_Yanbian-2017	1,04%	1,04%	0,97%	0,97%	0,84%	0,70%	1,11%	0,91%																							
10	PQ231071.1-TIGMIC4-YEZV-Ixodes_persulcatus-China:_Heilongjiang-2023	0,91%	0,91%	0,84%	0,84%	0,70%	0,56%	0,97%	0,77%	0,70%																						
11	LC735733.1-BT-1844-YEZV-Ixodes_persulcatus-Japan-2020	1,25%	1,25%	1,18%	1,18%	1,04%	0,97%	1,39%	1,18%	1,11%	0,97%																					
12	LC790682.1-BT-2155-YEZV-Ixodes_persulcatus-Japan-2021	1,11%	1,11%	1,04%	1,04%	0,91%	0,84%	1,25%	1,04%	0,97%	0,84%	0,14%																				
13	LC790679.1-BT-2135-YEZV-Ixodes_persulcatus-Japan-2021	0,97%	0,97%	0,91%	0,91%	0,77%	0,70%	1,11%	0,91%	0,84%	0,70%	0,42%	0,28%																			
14	NC_079100.1-HH003-2020-YEZV-Homo_sapiens-Japan-2020	1,18%	1,18%	1,11%	1,11%	0,97%	0,84%	1,25%	1,04%	0,97%	0,84%	0,84%	0,70%	0,70%																		
15	PV061582.1-Khabarovsk_2024-1-YEZV-Ixodes_persulcatus-Russia:_Khabarovsk-2024	1,18%	1,18%	1,11%	1,11%	0,97%	0,91%	1,32%	1,11%	1,04%	0,91%	1,18%	1,04%	0,91%	1,18%																	
16	ON563279.1-T-IM01-YEZV-Ixodes_persulcatus-China:_Hulunbuir_Inner_Mongolia-2021	1,11%	1,11%	1,04%	1,04%	0,91%	0,84%	1,25%	1,04%	0,97%	0,84%	1,11%	0,97%	0,84%	1,11%	0,35%																
17	PV061581.1-Chita_2024-1-YEZV-Ixodes_persulcatus-Russia:_Chita-2024	1,88%	1,88%	1,81%	1,81%	1,67%	1,60%	2,02%	1,81%	1,74%	1,60%	1,88%	1,74%	1,60%	1,88%	1,39%	1,32%															
18	PV770294-24-4-YEZV-Ixodes_persulcatus-Russia:_Kemerovo-2006	1,60%	1,60%	1,53%	1,53%	1,39%	1,25%	1,53%	1,39%	1,39%	1,25%	1,53%	1,53%	1,39%	1,53%	1,18%	1,11%	1,46%														
19	PV770291-7-YEZV-Homo_sapiens-Russia:_Kemerovo-2024	1,67%	1,67%	1,61%	1,61%	1,47%	1,33%	1,61%	1,47%	1,47%	1,33%	1,61%	1,61%	1,47%	1,61%	1,26%	1,19%	1,67%	0,21%													
20	PV770295-6-1-YEZV-Ixodes_persulcatus-Russia:_Kemerovo-2006	1,67%	1,67%	1,60%	1,60%	1,46%	1,32%	1,60%	1,46%	1,46%	1,32%	1,60%	1,60%	1,46%	1,46%	1,25%	1,18%	1,67%	0,35%	0,42%												
21	PV770296-6-4-YEZV-Ixodes_persulcatus-Russia:_Kemerovo-2006	1,67%	1,67%	1,60%	1,60%	1,46%	1,32%	1,60%	1,46%	1,46%	1,32%	1,60%	1,60%	1,46%	1,46%	1,25%	1,18%	1,67%	0,35%	0,42%	0,28%											
22	PV770292-143-YEZV-Ixodes_persulcatus-Russia:_Kemerovo-2023	1,60%	1,60%	1,53%	1,53%	1,39%	1,25%	1,53%	1,39%	1,39%	1,25%	1,53%	1,53%	1,39%	1,53%	1,18%	1,11%	1,60%	0,28%	0,35%	0,35%	0,35%										
23	PV770297-6-5-YEZV-Ixodes_persulcatus-Russia:_Kemerovo-2006	1,74%	1,74%	1,67%	1,67%	1,53%	1,39%	1,67%	1,53%	1,53%	1,39%	1,67%	1,67%	1,53%	1,67%	1,32%	1,25%	1,74%	0,42%	0,49%	0,49%	0,49%	0,42%									
24	LC621354.1-HH001-2019-YEZV-Homo_sapiens-Japan-2019	6,20%	6,20%	6,13%	6,13%	5,85%	5,85%	6,13%	6,34%	6,27%	6,27%	6,27%	6,27%	6,27%	6,27%	6,20%	6,13%	6,13%	6,20%	6,21%	6,27%	6,27%	6,20%	6,34%								
25	LC628645.1-HH009-2017-YEZV-Homo_sapiens-Japan-2017	6,48%	6,48%	6,41%	6,41%	6,13%	6,13%	6,41%	6,62%	6,55%	6,55%	6,55%	6,55%	6,55%	6,55%	6,48%	6,41%	6,41%	6,48%	6,49%	6,55%	6,55%	6,48%	6,62%	0,70%							
26	LC621360.1-HH011-2020-YEZV-Homo_sapiens-Japan-2020	6,13%	6,13%	6,20%	6,20%	6,06%	5,85%	6,34%	6,27%	6,20%	6,20%	6,13%	6,06%	6,20%	6,20%	5,99%	6,06%	6,06%	5,92%	5,93%	5,99%	5,85%	5,92%	6,06%	2,44%	2,58%						
27	PQ220362.1-MDJ2209-YEZV-Homo_sapiens-China:_Heilongjiang-2022	6,69%	6,69%	6,62%	6,62%	6,34%	6,34%	6,34%	6,69%	6,62%	6,62%	6,62%	6,62%	6,62%	6,62%	6,41%	6,48%	6,48%	6,41%	6,42%	6,48%	6,48%	6,41%	6,41%	2,51%	2,51%	3,27%					
28	PQ231070.1-TIGMIC3-YEZV-Ixodes_persulcatus-China:_Jilin-2019	6,75%	6,75%	6,69%	6,69%	6,41%	6,41%	6,41%	6,75%	6,62%	6,69%	6,69%	6,69%	6,69%	6,55%	6,48%	6,55%	6,41%	6,48%	6,42%	6,41%	6,41%	6,48%	6,48%	2,44%	2,44%	3,06%	1,74%				
29	ON563273.1-T-HLJ02-YEZV-Ixodes_persulcatus-China:_Heilongjiang-2021	6,75%	6,75%	6,69%	6,69%	6,41%	6,41%	6,41%	6,89%	6,62%	6,69%	6,69%	6,69%	6,69%	6,55%	6,48%	6,55%	6,55%	6,34%	6,35%	6,41%	6,41%	6,34%	6,34%	2,02%	2,02%	2,79%	1,46%	1,25%			
30	PV061579.1-Primorye_2024-2-YEZV-Ixodes_persulcatus-Russia:_Primorye-2024	4,81%	4,81%	4,74%	4,74%	4,60%	4,53%	4,67%	4,60%	4,60%	4,53%	4,74%	4,60%	4,53%	4,67%	4,46%	4,53%	4,53%	4,67%	4,68%	4,74%	4,74%	4,67%	4,81%	3,90%	4,04%	4,39%	3,55%	3,76%	3,55%		
31	PV770293-165-YEZV-Ixodes_persulcatus-Russia:_Kemerovo-2023	8,01%	8,01%	7,94%	7,94%	7,80%	7,87%	8,01%	8,15%	8,22%	7,94%	8,15%	8,08%	8,01%	8,36%	7,87%	7,80%	8,08%	7,80%	7,82%	7,87%	7,73%	7,80%	7,94%	8,43%	8,36%	8,36%	8,43%	8,22%	8,36%	8,15%	

B

		1	2	3	4	5	6	7	8	9	10	11	12	13	14	15	16	17	18	19	20	21	22	23	24	25	26	27	28	29	30	31
1	PX898225-560-YEZV-Ixodes_persulcatus-Russia:_Khabarovsk-2023																															
2	PX898228-662-YEZV-Ixodes_persulcatus-Russia:_Khabarovsk-2023	0,03%																														
3	PX898227-649-YEZV-Ixodes_persulcatus-Russia:_Khabarovsk-2023	0,03%	0,00%																													
4	PX898226-576-YEZV-Ixodes_persulcatus-Russia:_Khabarovsk-2023	0,03%	0,00%	0,00%																												
5	PQ220364.1-MDJ014-YEZV-Homo_sapiens-China:_Heilongjiang-2023	0,23%	0,21%	0,21%	0,21%																											
6	ON563278.1-T-IM01-YEZV-Ixodes_persulcatus-China:_Hulunbuir_Inner_Mongolia-2021	1,23%	1,21%	1,21%	1,21%	1,26%																										
7	PQ220367.1-MDJ2209-YEZV-Homo_sapiens-China:_Heilongjiang-2022	1,41%	1,39%	1,39%	1,39%	1,39%	1,64%																									
8	PQ231075.1-TIGMIC3-YEZV-Ixodes_persulcatus-China:_Jilin-2019	1,21%	1,18%	1,18%	1,18%	1,23%	1,49%	1,08%																								
9	PV061574.1-Primorye_2024-2-YEZV-Ixodes_persulcatus-Russia:_Primorye-2024	1,26%	1,23%	1,23%	1,23%	1,23%	1,51%	1,13%	1,13%																							
10	ON563272.1-T-HLJ02-YEZV-Ixodes_persulcatus-China:_Heilongjiang-2021	1,31%	1,28%	1,28%	1,28%	1,28%	1,49%	1,28%	1,28%	1,28%																						
11	ON563275.1-T-HLJ03-YEZV-Ixodes_persulcatus-China:_Heilongjiang-2021	1,67%	1,64%	1,64%	1,64%	1,69%	1,87%	1,90%	1,75%	1,69%	1,75%																					
12	ON563269.1-T-HLJ01-YEZV-Ixodes_persulcatus-China:_Heilongjiang-2021	1,49%	1,46%	1,46%	1,46%	1,51%	1,75%	1,62%	1,62%	1,57%	1,54%	1,21%																				
13	OR148887.1-YBQG1739-YEZV-Ixodes_persulcatus-China:_Yanbian-2017	1,31%	1,28%	1,28%	1,28%	1,34%	1,57%	1,54%	1,44%	1,34%	1,34%	0,98%	0,80%																			
14	PX898224-180-YEZV-Homo_sapiens-Russia:_Khabarovsk-2015	1,62%	1,59%	1,59%	1,59%	1,64%	1,87%	1,85%	1,75%	1,69%	1,64%	1,28%	1,16%	0,92%																		
15	PQ231076.1-TIGMIC4-YEZV-Ixodes_persulcatus-China:_Heilongjiang-2023	1,59%	1,57%	1,57%	1,57%	1,62%	1,85%	1,69%	1,72%	1,62%	1,62%	1,41%	1,28%	1,05%	1,31%																	
16	PV061577.1-Khabarovsk_2024-1-YEZV-Ixodes_persulcatus-Russia:_Khabarovsk-2024	1,75%	1,72%	1,72%	1,72%	1,77%	1,85%	1,87%	1,77%	1,77%	1,82%	1,64%	1,51%	1,23%	1,54%	1,57%																
17	LC735732.1-BT-1844-YEZV-Ixodes_persulcatus-Japan-2020	1,49%	1,46%	1,46%	1,46%	1,51%	1,75%	1,67%	1,57%	1,51%	1,51%	1,36%	1,28%	1,05%	1,36%	1,28%	1,46%															
18	LC790681.1-BT-2155-YEZV-Ixodes_persulcatus-Japan-2021	1,49%	1,46%	1,46%	1,46%	1,51%	1,75%	1,67%	1,57%	1,51%	1,51%	1,36%	1,28%	1,05%	1,36%	1,28%	1,46%	0,05%														
19	NC079098.1-HH003-2020-YEZV-Homo_sapiens-Japan-2020	1,54%	1,51%	1,51%	1,51%	1,57%	1,80%	1,67%	1,57%	1,57%	1,51%	1,41%	1,34%	1,10%	1,41%	1,34%	1,51%	0,82%	0,82%													
20	PV061576.1-Chita_2024-1-YEZV-Ixodes_persulcatus-Russia:_Chita-2024	1,75%	1,72%	1,72%	1,72%	1,77%	1,93%	1,87%	1,87%	1,82%	1,77%	2,13%	1,87%	1,72%	1,93%	2,00%	2,11%	1,95%	1,95%	2,05%												
21	LC621353.1-HH001-2019-YEZV-Homo_sapiens-Japan-2019	2,05%	2,03%	2,03%	2,03%	2,08%	2,21%	2,23%	2,18%	2,08%	2,08%	2,34%	2,16%	1,98%	2,18%	2,26%	2,46%	2,21%	2,21%	2,16%	1,95%											
22	LC790678.1-BT-2135-YEZV-Ixodes_persulcatus-Japan-2021	2,36%	2,34%	2,34%	2,34%	2,39%	2,52%	2,54%	2,49%	2,39%	2,39%	2,70%	2,52%	2,34%	2,59%	2,62%	2,77%	2,52%	2,52%	2,54%	2,26%	1,05%										
23	LC621359.1-HH011-2020-YEZV-Homo_sapiens-Japan-2020	2,39%	2,36%	2,36%	2,36%	2,41%	2,57%	2,62%	2,52%	2,41%	2,41%	2,77%	2,59%	2,41%	2,46%	2,64%	2,72%	2,54%	2,54%	2,59%	2,16%	1,36%	1,69%									
24	OQ994742.1-HH009-2017-YEZV-Homo_sapiens-Japan-2017	2,64%	2,62%	2,62%	2,62%	2,67%	2,82%	2,82%	2,77%	2,67%	2,62%	3,08%	2,85%	2,72%	2,77%	2,95%	3,00%	2,90%	2,90%	2,90%	2,46%	1,72%	2,03%	1,18%								
25	PX898220-24-4-YEZV-Ixodes_persulcatus-Russia:_Kemerovo-2006	7,45%	7,42%	7,42%	7,42%	7,42%	7,70%	7,60%	7,60%	7,55%	7,65%	7,86%	7,63%	7,57%	7,75%	7,78%	7,78%	7,88%	7,88%	7,96%	7,55%	7,55%	7,63%	7,80%	7,91%							
26	PX898223-7-YEZV-Homo_sapiens-Russia:_Kemerovo-2024	7,57%	7,55%	7,55%	7,55%	7,55%	7,83%	7,73%	7,73%	7,68%	7,78%	7,98%	7,75%	7,70%	7,83%	7,96%	7,75%	7,96%	7,96%	8,09%	7,68%	7,68%	7,70%	7,88%	7,98%	0,39%						
27	PX898221-143-YEZV-Ixodes_persulcatus-Russia:_Kemerovo-2023	7,63%	7,60%	7,60%	7,60%	7,60%	7,80%	7,78%	7,73%	7,73%	7,78%	8,01%	7,80%	7,75%	7,93%	8,01%	7,91%	8,06%	8,06%	8,14%	7,73%	7,73%	7,80%	7,93%	8,04%	0,62%	0,69%					
28	PX898217-6-1-YEZV-Ixodes_persulcatus-Russia:_Kemerovo-2006	7,45%	7,42%	7,42%	7,42%	7,42%	7,65%	7,60%	7,60%	7,60%	7,65%	7,88%	7,63%	7,63%	7,75%	7,83%	7,73%	7,88%	7,88%	7,96%	7,55%	7,55%	7,63%	7,80%	7,91%	0,49%	0,56%	0,64%				
29	PX898218-6-4-YEZV-Ixodes_persulcatus-Russia:_Kemerovo-2006	7,50%	7,47%	7,47%	7,47%	7,47%	7,65%	7,65%	7,65%	7,65%	7,70%	7,91%	7,68%	7,68%	7,80%	7,88%	7,78%	7,88%	7,88%	8,01%	7,60%	7,60%	7,68%	7,86%	7,96%	0,46%	0,54%	0,62%	0,28%			
30	PX898219-6-5-YEZV-Ixodes_persulcatus-Russia:_Kemerovo-2006	7,32%	7,29%	7,29%	7,29%	7,29%	7,52%	7,47%	7,47%	7,47%	7,52%	7,73%	7,50%	7,50%	7,63%	7,65%	7,60%	7,75%	7,75%	7,83%	7,42%	7,42%	7,50%	7,68%	7,78%	0,41%	0,54%	0,62%	0,28%	0,36%		
31	PX898222-165-YEZV-Ixodes_persulcatus-Russia:_Kemerovo-2023	9,47%	9,45%	9,45%	9,45%	9,35%	9,68%	9,53%	9,45%	9,47%	9,55%	9,70%	9,50%	9,63%	9,63%	9,68%	9,53%	9,86%	9,86%	9,63%	9,40%	9,14%	9,55%	9,35%	9,58%	9,01%	9,09%	9,09%	8,96%	9,04%	8,91%	

C

		1	2	3	4	5	6	7	8	9	10	11	12	13	14	15	16	17	18	19	20	21	22	23	24	25	26	27	28	29
1	OR148889.1-YBQG1739-YEZV-Ixodes_persulcatus-China:_Yanbian-2017																													
2	ON563274.1-T-HLJ03-YEZV-Ixodes_persulcatus-China:_Heilongjiang-2021	0,99%																												
3	PX904398-180-YEZV-Homo_sapiens-Russia:_Khabarovsk-2015	0,92%	0,92%																											
4	PQ231078.1-TIGMIC4-YEZV-Ixodes_persulcatus-China:_Heilongjiang-2023	1,15%	1,15%	1,02%																										
5	ON563268.1-T-HLJ01-YEZV-Ixodes_persulcatus-China:_Heilongjiang-2021	1,01%	1,01%	0,97%	1,09%																									
6	LC735731.1-BT-1844-YEZV-Ixodes_persulcatus-Japan-2020	1,32%	1,36%	1,17%	1,37%	1,38%																								
7	LC790680.1-BT-2155-YEZV-Ixodes_persulcatus-Japan-2021	1,27%	1,30%	1,16%	1,35%	1,32%	0,06%																							
8	LC790677.1-BT-2135-YEZV-Ixodes_persulcatus-Japan-2021	1,25%	1,25%	1,14%	1,30%	1,27%	0,84%	0,79%																						
9	NC079099.1-HH003-2020-YEZV-Homo_sapiens-Japan-2020	1,17%	1,17%	1,06%	1,18%	1,19%	0,77%	0,72%	0,48%																					
10	ON811933.1-TIGMIC3-YEZV-Ixodes_persulcatus-China:_Jilin-2019	1,74%	1,67%	1,61%	1,78%	1,65%	1,67%	1,61%	1,60%	1,52%																				
11	PQ220372.1-MDJ2209-YEZV-Homo_sapiens-China:_Heilongjiang-2022	1,67%	1,54%	1,56%	1,74%	1,63%	1,58%	1,52%	1,50%	1,43%	1,17%																			
12	PV061569.1-Primorye_2024-2-YEZV-Ixodes_persulcatus-Russia:_Primorye-2024	1,67%	1,63%	1,54%	1,72%	1,58%	1,63%	1,58%	1,56%	1,49%	1,25%	1,19%																		
13	ON563277.1-T-IM01-YEZV-Ixodes_persulcatus-China:_Hulunbuir_Inner_Mongolia-2021	1,85%	1,74%	1,72%	1,87%	1,69%	1,71%	1,65%	1,67%	1,60%	1,54%	1,49%	1,50%																	
14	PV061572.1-Khabarovsk_2024-1-YEZV-Ixodes_persulcatus-Russia:_Khabarovsk-2024	1,82%	1,74%	1,69%	1,83%	1,61%	1,67%	1,61%	1,63%	1,56%	1,50%	1,45%	1,43%	0,70%																
15	ON563271.1-T-HLJ02-YEZV-Ixodes_persulcatus-China:_Heilongjiang-2021	1,67%	1,60%	1,50%	1,72%	1,54%	1,56%	1,50%	1,41%	1,38%	1,36%	1,30%	1,32%	1,39%	1,39%															
16	PX904391-560-YEZV-Ixodes_persulcatus-Russia:_Khabarovsk-2023	1,83%	1,71%	1,71%	1,85%	1,78%	1,78%	1,72%	1,67%	1,60%	1,49%	1,45%	1,52%	1,67%	1,63%	1,52%														
17	PX904392-576-YEZV-Ixodes_persulcatus-Russia:_Khabarovsk-2023	1,83%	1,71%	1,71%	1,85%	1,78%	1,78%	1,72%	1,67%	1,60%	1,49%	1,45%	1,52%	1,67%	1,63%	1,52%	0,00%													
18	PX904390-649-YEZV-Ixodes_persulcatus-Russia:_Khabarovsk-2023	1,96%	1,83%	1,83%	1,98%	1,91%	1,91%	1,85%	1,80%	1,71%	1,61%	1,58%	1,65%	1,80%	1,76%	1,63%	0,29%	0,29%												
19	PX904393-662-YEZV-Ixodes_persulcatus-Russia:_Khabarovsk-2023	1,83%	1,71%	1,71%	1,85%	1,78%	1,78%	1,72%	1,67%	1,60%	1,49%	1,45%	1,52%	1,67%	1,63%	1,52%	0,15%	0,15%	0,15%											
20	PQ220369.1-MDJ014-YEZV-Homo_sapiens-China:_Heilongjiang-2023	1,80%	1,71%	1,67%	1,81%	1,74%	1,74%	1,69%	1,63%	1,56%	1,52%	1,45%	1,49%	1,67%	1,60%	1,49%	0,33%	0,33%	0,48%	0,33%										
21	LC621358.1-HH011-2020-YEZV-Homo_sapiens-Japan-2020	2,90%	2,75%	2,73%	3,02%	2,83%	2,84%	2,83%	2,70%	2,66%	2,62%	2,44%	2,61%	2,68%	2,66%	2,55%	2,66%	2,66%	2,77%	2,66%	2,62%									
22	OQ994741.1-HH009-2017-YEZV-Homo_sapiens-Japan-2017	2,92%	2,73%	2,73%	2,96%	2,81%	2,90%	2,84%	2,72%	2,68%	2,68%	2,49%	2,61%	2,75%	2,72%	2,59%	2,64%	2,64%	2,75%	2,64%	2,64%	1,01%								
23	LC621352.1-HH001-2019-YEZV-Homo_sapiens-Japan-2019	2,79%	2,62%	2,70%	2,94%	2,77%	2,79%	2,73%	2,57%	2,57%	2,53%	2,53%	2,49%	2,61%	2,57%	2,46%	2,59%	2,59%	2,70%	2,59%	2,59%	1,89%	1,89%							
24	PV061571.1-Chita_2024-1-YEZV-Ixodes_persulcatus-Russia:_Chita-2024	3,08%	3,06%	2,94%	3,15%	3,12%	3,01%	2,95%	2,90%	2,86%	2,88%	2,75%	2,73%	2,95%	2,88%	2,68%	2,99%	2,99%	3,10%	2,99%	2,95%	2,95%	2,86%	2,81%						
25	PX904396-143-YEZV-Ixodes_persulcatus-Russia:_Kemerovo-2023	4,68%	4,73%	4,72%	4,96%	4,88%	4,86%	4,84%	4,79%	4,72%	4,64%	4,37%	4,57%	4,57%	4,48%	4,51%	4,77%	4,77%	4,88%	4,77%	4,66%	4,61%	4,73%	4,68%	4,83%					
26	PX904394-6-5-YEZV-Ixodes_persulcatus-Russia:_Kemerovo-2006	4,70%	4,75%	4,73%	4,98%	4,90%	4,88%	4,86%	4,81%	4,73%	4,66%	4,37%	4,57%	4,62%	4,53%	4,53%	4,83%	4,83%	4,94%	4,83%	4,72%	4,62%	4,75%	4,70%	4,81%	0,37%				
27	PX904395-24-4-YEZV-Ixodes_persulcatus-Russia:_Kemerovo-2006	4,73%	4,77%	4,77%	5,01%	4,90%	4,92%	4,90%	4,84%	4,77%	4,73%	4,44%	4,66%	4,70%	4,61%	4,61%	4,83%	4,83%	4,94%	4,83%	4,72%	4,70%	4,75%	4,77%	4,92%	0,48%	0,51%			
28	PX904399-7-YEZV-Homo_sapiens-Russia:_Kemerovo-2024	4,66%	4,70%	4,70%	4,94%	4,82%	4,84%	4,82%	4,77%	4,70%	4,62%	4,33%	4,55%	4,62%	4,49%	4,49%	4,75%	4,75%	4,86%	4,75%	4,60%	4,62%	4,68%	4,70%	4,81%	0,48%	0,48%	0,37%		
29	PX904397-165-YEZV-Ixodes_persulcatus-Russia:_Kemerovo-2023	10,1%	10,2%	10,1%	10,3%	10,2%	10,2%	10,1%	10,2%	10,0%	10,1%	9,9%	9,9%	10,1%	10,1%	9,7%	10,2%	10,2%	10,2%	10,1%	10,2%	9,9%	9,9%	10,0%	9,8%	10,1%	10,1%	10,3%	10,1%	

Appendix Figure 2. Estimates of evolutionary divergence between nucleotide sequences of Yezo virus A) S segment, B) M segment, C) L segment. The percentages of base differences per site between sequences are shown. The analysis was conducted using MEGA12.
